# Differences in Pubertal Curve Progression among Females with Adolescent Idiopathic Scoliosis Using Pregnenolone Therapy: A Retrospective Case-Controlled Series

**DOI:** 10.3390/jcm13030788

**Published:** 2024-01-30

**Authors:** Mark W. Morningstar, Brooke DuRussel

**Affiliations:** 1 Natural Wellness & Pain Relief Center, Grand Blanc, MI 48423, USA; 2 Lyman Briggs College and the Department of Natural Science, Michigan State University, East Lansing, MI 48824, USA; duruss11@msu.edu

**Keywords:** hormone, scoliosis, spine

## Abstract

**Background**: Differences in hormone metabolism have been observed in children with adolescent idiopathic scoliosis. These differences have been offered as underlying reasons for rapid curve progression during puberty. This study retrospectively compared two groups of females with a history of adolescent idiopathic scoliosis. They were seen for initial presentation prior to menarche, or within 2 months after menarche, and they were followed up 1 year after first menarche. **Methods**: All patients in both groups underwent baseline salivary hormone testing to identify any hormone imbalances. The control group was composed of females with curves between 10 and 25 degrees and maintained an observation-only management strategy. The treatment group showed baseline curve measurements ranging from 10 to 23 degrees, and additionally took pregnenolone daily for 12 months. **Results**: At one-year follow-up, the treatment group showed curve measurements ranging from 13 to 24 degrees, while the control group ranged from 16 to 29 degrees (*p* < 0.05). **Conclusions**: The study showed that adolescent females taking pregnenolone daily for low progesterone had reduced scoliosis curve progression over 1 year compared to controls.

## 1. Introduction

Adolescent idiopathic scoliosis refers to a condition marked by a spinal curvature measuring 10° or more, as determined by Cobb’s angle on radiographic examination [1]. The prevalence of this condition is estimated to range from 0.47% to 5.2% among individuals aged 0–17 in the United States, with a subsequent increase to 8% in the adult population [1]. Treatment approaches for adolescent idiopathic scoliosis depend on the initial curvature magnitude, as outlined in the 2016 SOSORT treatment guidelines [1]. These guidelines recommend exercises for curves between 10 and 15 degrees, while curves ranging from 15 to 50 degrees should be managed with exercises and/or bracing, contingent on the patient’s growth stage. Curves exceeding 50° typically warrant surgical interventions like spinal fusion surgery [2].

Despite these treatment guidelines addressing the spinal curvature, they do not encompass certain non-spinal comorbidities commonly associated with this condition, such as the differences in levels of hormones like vitamin D3 [3], leptin, ghrelin [4], FSH, LH, estrogen, progesterone [5], and melatonin [6]. These and other similar observations have contributed to a more comprehensive, hypothetical understanding of scoliosis etiopathogenesis [7].

Building upon these earlier observations in idiopathic scoliosis, the present study delves into specific hormone differences in individuals with scoliosis. Notably, progesterone exerts a significant influence on the motor memory centers of the brain, such as the hippocampus and thalamus, through synaptic plasticity [8]. Progesterone also serves to signal peak bone mass development, which may also contribute to the observed differences in bone mineral density among adolescent idiopathic scoliosis patients [9]. Progesterone is derived from pregnenolone, which is also considered a neurotransmitter, acting centrally on N-methyl-D-aspartate (NMDA) receptors to modulate glutaminergic activity [10]. This pathway enables pregnenolone to exert positive influences on neuroplasticity, which may also be important in idiopathic scoliosis given the cerebellar/hindbrain component of scoliosis etiology [7].

This study reports the observations of scoliosis Cobb angle and salivary progesterone levels in scoliosis patients who were given pregnenolone for low progesterone, as compared to scoliosis patients who declined pregnenolone use or were otherwise not candidates. Pregnenolone is an immediate metabolic precursor to progesterone and is itself produced intrinsically from cholesterol. Pregnenolone has been shown to enhance locomotor activity, learning, and memory, and to promote neuron survival [11]. Considering the proposed neurological mechanisms involved in idiopathic scoliosis [12], as well as its ability to improve progesterone levels [13], the current study aims to retrospectively assess the data from a cohort of female patients who were prescribed pregnenolone for low salivary-progesterone values in relation to their scoliosis history.

## 2. Materials and Methods

### 2.1. Study Design

This research adhered to a retrospective case–control study design in line with the STROBE guidelines. The study aimed to observe any differences in the amount of pubertal curve progression in a cohort of patients with a history of idiopathic scoliosis if they concurrently took pregnenolone supplementation specifically for low progesterone levels. Historical data were considered.

### 2.2. Participants

A consecutive sampling method was employed, involving all patient records from a multidisciplinary medical clinic in Grand Blanc, MI, USA, from January 2019 to November 2022. Inclusion criteria for the present study were comprised of the following: (1) patients with a history of adolescent idiopathic scoliosis, (2) biological females, (3) either pre-menses or had their first menstrual period with the prior 2 months, and (4) completed a salivary hormone test and were subsequently given pregnenolone as part of their clinical management. A total of 14 patient files meeting these criteria were consecutively selected and included in the treatment group. A second group of scoliosis patient files were selected, using the same inclusion criteria except these patients did not start pregnenolone therapy. Consecutive sampling was also used until 13 files were collected. Data collection exclusively involved female patients for both groups to ensure consistency in reference progesterone levels, and males were excluded for homogeneity.

### 2.3. Variables

Non-identifying data for both groups included age, salivary progesterone levels, and radiographic Cobb angle measurements at baseline and 1 year. Cobb angle measurements were drawn from the superior endplate of the upper, most-tilted vertebra and the inferior endplate of the lowest, most-tilted vertebra. The measurement was derived from the angle created by the intersection of two lines drawn perpendicular from each endplate. Salivary progesterone levels were obtained through Labrix (St. Charles, IL, USA) (http://www.doctorsdata.com, accessed on 10 October 2023) due to its noninvasive nature, making it especially suitable for adolescent patients. Salivary progesterone has demonstrated a consistent correlation with serum progesterone values [14]; therefore, it was chosen for its ease of collection, particularly for younger patients. Salivary progesterone values are reported in picograms per milliliter. Confounding factors include patients within each group whose curves warranted participation in some type of physical treatment (i.e., scoliosis-specific exercises, bracing). A history of scoliosis also presents as a potential confounding variable, since the pregnenolone therapy was focused on treating low progesterone values, not idiopathic scoliosis. Patients who began pregnenolone supplementation were placed on either 10 mg, 20 mg, or 30 mg once every evening. This was administered orally in 10 mg tablets. The initial dose was based on baseline patient weight as well as the deficit in each patient’s baseline progesterone value relative to the low end of the reference range published by the laboratory.

### 2.4. Bias

Given the retrospective nature of the current study, selection bias is of primary concern. If an effort to minimize this bias, matching criteria were applied to ensure comparability between cases and controls. The use of the primary outcome variables, radiographic Cobb angle measurements, and salivary progesterone levels also minimize the risk of recall bias and interviewer bias.

### 2.5. Statistical Analysis

Statistical analyses were conducted in R Studio [15], with additional data averages derived using Excel, to determine whether relationships existed for a multitude of variables. The first hypothesis tested whether the pregnenolone dosage had an impact on the change in progesterone levels between the treatment group and the control group. One-sample *t*-tests with 95% confidence intervals were used to look at the entire group of participants and then the treatment group only. *p*-values were used to determine whether the relationship was of statistical significance. Welch’s *t*-test was used with a 95% confidence interval for the entire group’s *t*-test. The dependent variable was the change in salivary progesterone levels. The independent variable was the dosage of pregnenolone or lack thereof. The alternative hypothesis postulated that there would be no difference in the average change in salivary progesterone levels between the groups. The relationship was then furthered to see if there was a dose–response relationship between the amount of pregnenolone administered and the change in progesterone levels upon one year of follow up. Dose–response analyses of the treatment group’s dosages were performed along with another dose–response analysis with all the participants in R-Studio Version 4.2.1 using the drm() function [15]. A summary plot of the dose–response analysis was also made for both analyses. Log-logistic analyses under three parameters were used to determine the *p*-values for the dose–response relationship for the treatment group, and four parameters were used for the entire group of participants.

The second relationship tested was whether all participants, regardless of therapy, experienced a difference in curve progression based on their age. Scatterplots with age as the independent variable and change in curve progression were made in Excel. Three different graphs were made using Excel Version 2312 and R Studio Version 4.2.1 [15]: all participants, treatment group only, and control group only. Trendlines with their corresponding linear equations and R-squared values were made to visualize and quantify the potential relationship. Using R-Studio, a histogram of the change in Cobb angle was also made to determine if the variable was normally distributed amongst the participants. Correspondingly, in R-Studio, an asymptotic one-sample Kolmogorov–Smirnov test was used to test the normal distribution of the change in Cobb angle in all the participants. A scatterplot was also created in R-Studio with age as the independent variable and change in Cobb angle as the dependent variable. A linear regression analysis in R-Studio was performed on the graph to find the *p*-value, F-statistic, R-squared value, degrees of freedom, t-value of the slope, t-value of the intercept, standard error of the y-intercept, standard error or the slope, and formula for the linear regression line. The values were compared against the standard error and linear regression formula found in Excel to verify results.

Another relationship tested in R-Studio was whether the dosage of pregnenolone alters the course of curve progression within the treatment group. Dose–response analyses of the treatment group’s dosages were performed along with another dose–response analysis with all the participants in R-Studio using the drm() function [15]. A summary plot of the dose–response analysis to the change in Cobb angle was also made for both analyses. Log-logistic analyses under three parameters were used to determine the *p*-values for the dose–response relationship for the treatment group, and four parameters were used for the entire group of participants.

To test for the confounding variables of having scoliosis initially and initial progesterone levels, ANCOVA tests were performed using R-Studio for each of the variables separately [15]. ANOVA tests were first performed on the variables to determine if the co-variates of initial Cobb angle (the presence of scoliosis) and progesterone levels met the criteria for an ANCOVA test. The independent variable was the pregnenolone dosage, and *p*-values were used to determine if the variables were independent. Following the ANOVA tests, ANCOVA analyses were performed using the change in Cobb angle as the response variable, the pregnenolone dosage as the treatment variable, and the initial Cobb angle and progesterone levels as the covariates separately. A type III sum of squares was used for the analysis to obtain the *p*-values using R Studio [15].

## 3. Results

**Figure 1:**
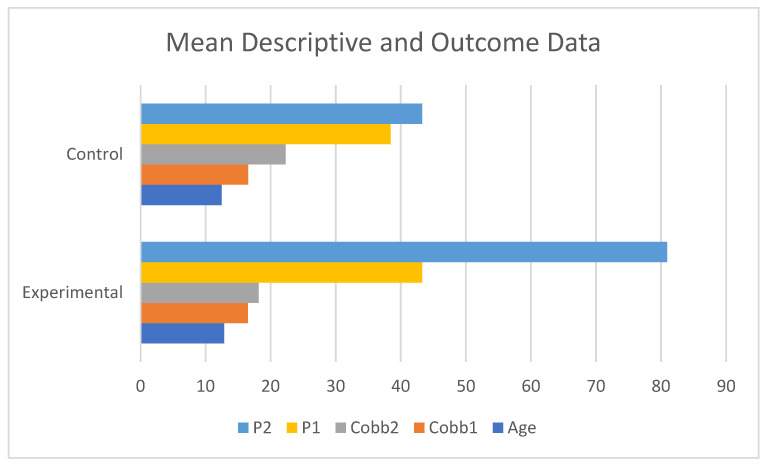
Mean descriptive and outcome data.

The mean age of the treatment group was 12.83 years, with a range of 10–15 years. The control group had a mean age of 12.42 years, and a range of 10–15 years. The average baseline Cobb angles for the experimental group and control group were 16.5 and 16.5 degrees, respectively, with 4.03 and 4.45 degrees of standard deviation for the treatment and control groups, respectively. The average baseline height was 154.71 cm for the treatment group and 148.46 cm for the control group. At 1 year, these values increased to 156.43 cm and 149.54 cm, respectively. Figure 1 shows a comparison of demographic and outcome data for age, initial and final Cobb angles, and initial and final serum progesterone levels between the groups.

At 1-year follow-up, the treatment group’s average Cobb angle was 18.14 degrees ±3.44, while the control group’s average was 22.31 degrees ±4.32. The mean difference in Cobb angle at 1 year for the treatment group was 1.6 degrees, while the control group’s mean difference was 5.8 degrees.

**Figure 2:**
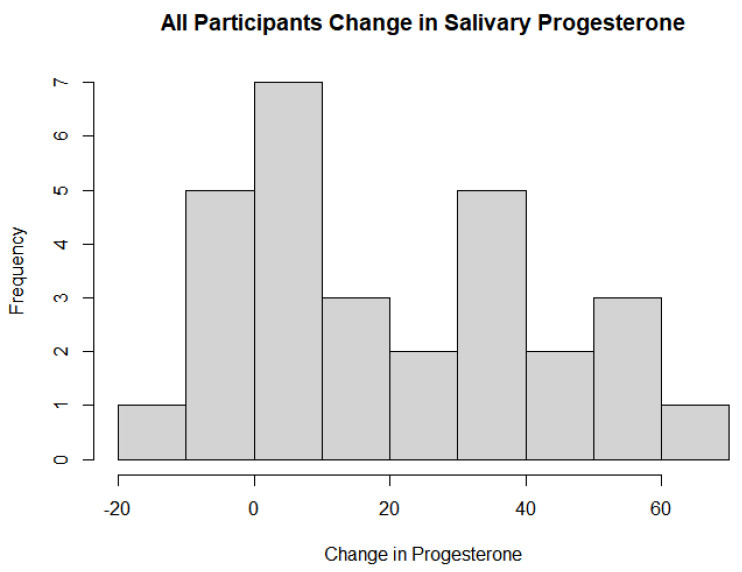
All participants change in salivary progesterone levels following pregnenolone therapy.

**Figure 3:**
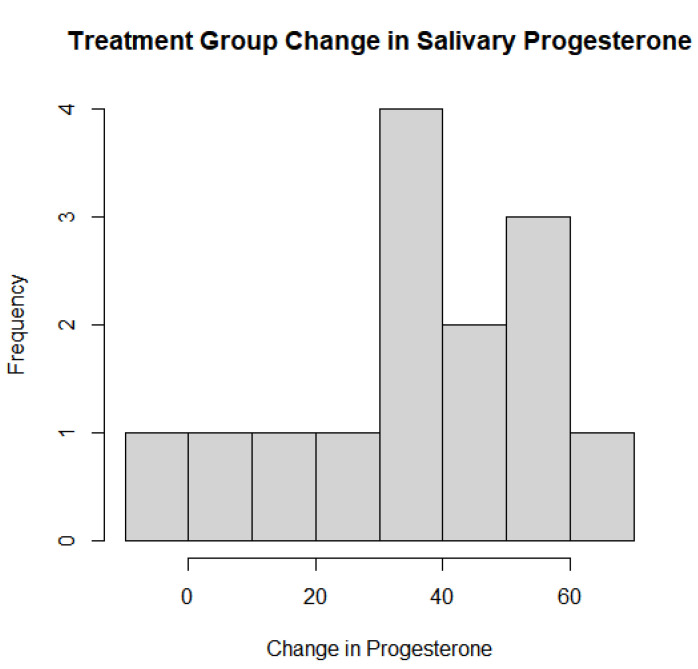
Treatment group change in salivary progesterone levels following pregnenolone therapy.

**Figure 4:**
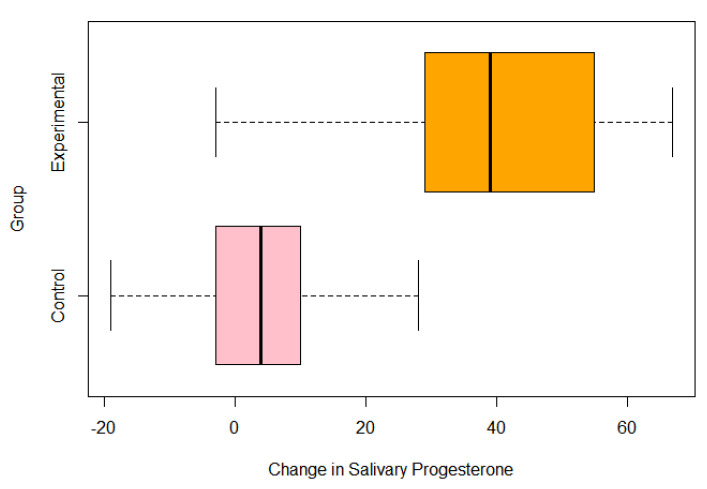
Boxplot of change in salivary progesterone across groups.

The baseline and 1-year salivary progesterone levels in the treatment group were averages of 43.3 pg/mL and 80.9 pg/mL, respectively. The averages for the control group were 38.5 pg/mL and 43.3 pg/mL. For the Welch’s *t*-test analyzing whether there was a statistically significant effect on salivary progesterone levels depending on the usage of pregnenolone therapy, there was extremely strong evidence that the pregnenolone therapy influenced the salivary hormone levels with a *p*-value of 0.00004739. The 95% confidence interval was between −46.19 and −19.40 to indicate that the mean between the groups was not equal to 0. The one-sample *t*-test of the treatment group based on dosage and its effects on salivary progesterone levels also showed extremely strong evidence that the pregnenolone therapy increased the salivary progesterone levels with a *p*-value of 0.0000121 and a 95% confidence interval between 25.73 and 49.55. As the mean change was much higher than 0 for this confidence interval while the other 95% confidence interval testing the means between the experimental and treatment groups was less than 0, this indicates that pregnenolone therapy did have a substantial impact on improving the salivary progesterone levels more so than not taking any pregnenolone at all. The histograms of the entire group of participants as well as the treatment group only with their respective changes in salivary progesterone levels are shown in Figure 2 and Figure 3 below. Figure 4 details the mean and quartile ranges of the change in progesterone between the experimental and control group (note that the colors in Figure 4 bear no significant meaning).

**Figure 5:**
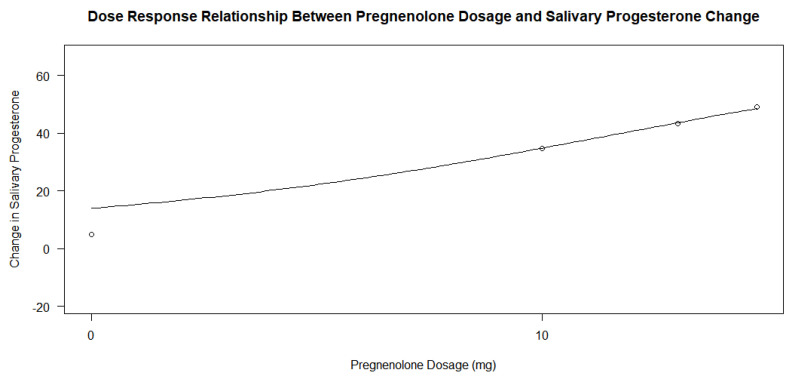
Dose–response relationship between pregnenolone dosage and salivary progesterone change.

**Figure 6:**
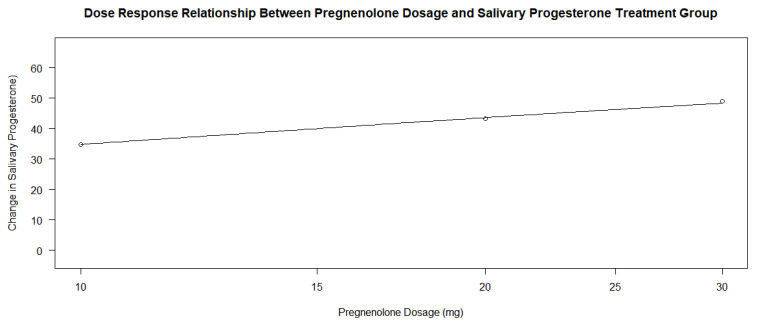
Dose–response relationship between pregnenolone dosage and salivary progesterone change in treatment group only.

When determining whether the pregnenolone dosage showed a dose–response relationship with the amount of salivary progesterone change, there was an insignificant relationship between the dosage and the change based on the *p*-value alone; however, this may be due to the relationship between the treatment group participants only. When looking at the analysis of the entire group of participants, the *p*-values of the four parameters in the drm() test in R yielded values of 0.8300, 0.3259, 0.7877, and 0.9297. On initial conclusion, it would appear that the dosage had no effect on progesterone levels following treatment. However, when analyzing the graphs in Figure 5 and Figure 6, and the *p*-values of the dose–response relationship in the treatment group alone, it would appear that the treatment group experienced a much larger increase in salivary progesterone compared to the control group. The control group showed an average change of 4.85, while each group’s change in the treatment subsets was greater than 30 (34.80, 43.33, and 49, respectively). The highest change in the experiment group was 67, while the highest change in the control was 28 (a number lower than only three of the changes in the experimental group). When the dose–response relationship for the treatment group only was conducted using the drm() function in R Studio and a log-logistic function with three parameters, the *p*-values obtained were 0.8738, 0.9812, and 0.8832. These massive *p*-values show that the dosage of pregnenolone in the treatment group had no effect on the change in salivary progesterone levels. Any pregnenolone dosage was sufficient to cause a change in the salivary progesterone levels. Since the treatment group accounts for a little over half of the participants in the study (14 out of 27), the *p*-values analyzing the groups together are skewed to be larger. In this case, the argument for the dose–response relationship cannot be made based on *p*-values alone when comparing the groups simultaneously. The pregnenolone dosage did change the levels of salivary hormones of progesterone between the control group and inclusive subsets of the treatment groups.

**Figure 7:**
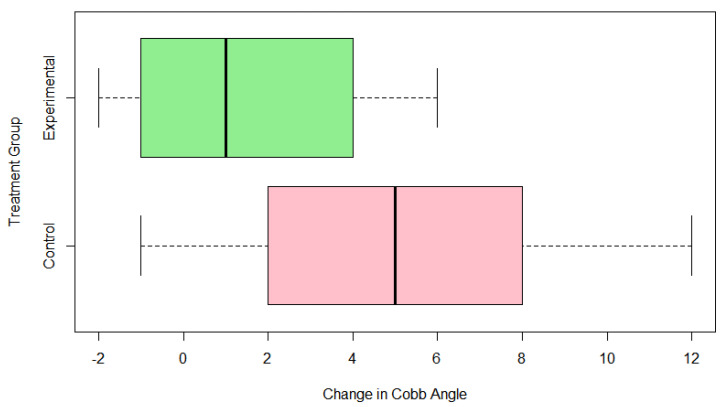
Boxplot of change in cobb angle across groups.

Upon investigating whether the pregnenolone changed the development of the Cobb angle, one-sample *t*-tests for each group separately showed that the Cobb angle changes were of low statistical significance in the treatment group (*p* = 0.05185), but very statistically significant in the control group (*p* = 0.0001949). The *t*-test was set with mu = 0, the average change in the treatment group was 1.64 degrees, and the average change in the control group was 5.77 degrees. In looking at the *p*-values and the average changes, the treatment group showed a change that was not statistically significant from zero. In this instance, being closer to zero indicates a smaller curve progression (a more positive number from zero indicates that the curve has progressed). The control group showed an average change in Cobb angle of 5.77 degrees with a *p*-value of 0.0001949. This leads to the result that the control group had statistically significant evidence that the curves did progress beyond 0 to become worse without the pregnenolone therapy. The pregnenolone therapy resulted in a smaller curve progression compared to those not treated with the pregnenolone therapy. From the Welch’s *t*-test conducted in R-Studio to analyze the relationship between a difference in curve progression between the treatment and control groups, it was found that there was a statistically significant difference in curve angle progression based off the treatment of pregnenolone given to the two groups. When conducting a Welch’s *t*-test across the groups, a *p*-value of 0.005378 was given for a 95% confidence interval of (1.355765–6.896982). Since the *p*-value of 0.005378 shows statistically significant results, the experimental group receiving the pregnenolone experienced a smaller progression of their Cobb angle following treatment. There is strong evidence to suggest that the experimental group exhibited a smaller progression in their Cobb angle from the treatment. As zero is not included in the 95% confidence interval, this strengthens the argument that the treatment caused a decreased curve progression compared to the control group. From the Excel and R Studio analyses, the following information was produced. The experimental group’s mean difference in Cobb angle was 1.642 degrees. The control group’s mean difference in Cobb angle was 5.769 degrees. The standard deviation in Cobb angle difference for the experimental group was 2.767 degrees. The standard deviation in Cobb angle difference for the control group was 3.786 degrees. The Welch’s *t*-test t-value was 3.0899, which was positive because there was a smaller mean for the experimental group to indicate a smaller Cobb angle progression. Figure 7 highlights a boxplot of the mean outcomes of Cobb angle change between the two groups, with the experimental group having the lower average Cobb angle change and quartile range (note that colors bear not significant meaning).

**Figure 8:**
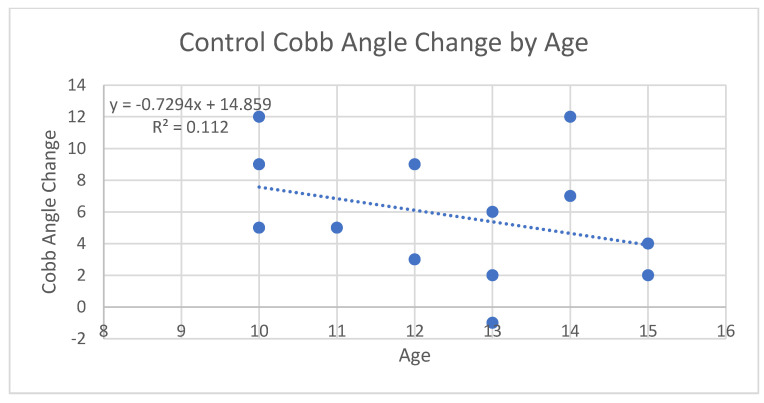
Control group cobb angle data.

**Figure 9:**
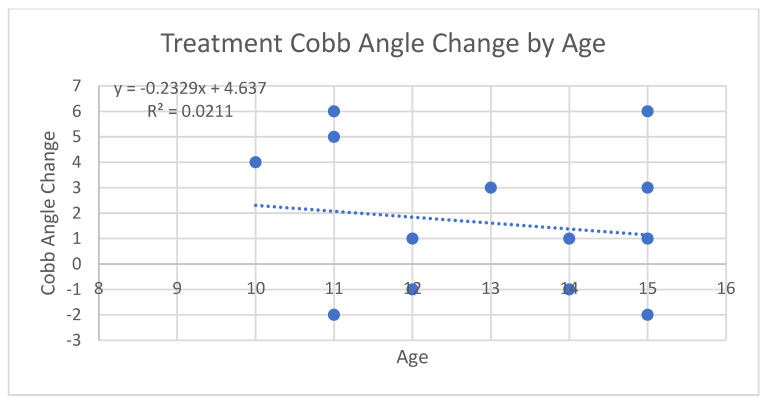
Treatment group cobb angle data.

**Figure 10:**
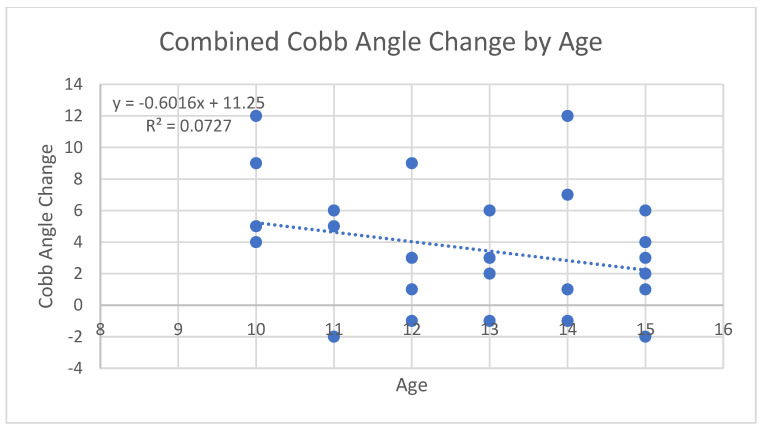
Intergroup cobb angle data comparison.

After comparing data for progesterone levels and Cobb angle measurements, we analyzed the potential relationship between age at baseline and amount of curve progression among both groups. It has been reported previously that younger ages of onset may be correlated with a higher risk of curve progression [1]. To evaluate this, scatterplots were created for each group as shown in Figure 8 and Figure 9, as well as one for both groups combined as shown in Figure 10. When analyzing the trendline for the control group, the trendline had a negative slope of −0.7294 with an R squared value of 0.112. The treatment group had a negative trendline with a slope of −0.2329 with an R squared value of 0.0211, and the combined group showed a negative trendline with a slope of −0.6016 with an R squared value of 0.0727. It was found that both groups combined also had a *p*-value of 0.4496. Statistical analysis was completed in R Studio as well as Excel. After completing data analysis for age of onset and its relation to Cobb angle curve progression, the relationship is not statistically significant. Observing the scatterplots of all the groups, the graphs all exhibit negative slopes but critically low R squared values (0.0211, 0.112, and 0.0727, respectively). For the combined groups of all 27 participants, the curve progression (change in Cobb angle) tended to decrease as age increased; however, this was not the case for all of the points, as shown in Figure 10. There were some outliers in the young age group who had an increase in Cobb angle that was as high as that of the 15 year old group, but the trend tended to show no decreases in Cobb angle with younger participant ages upon diagnosis. As the age of onset increased in the treatment group (age 11 is the first instance of a decrease in Cobb angle), there was a greater likelihood of decreasing patient Cobb angle. This was not true in some cases, and this could be attributed to compliance with other aspects of treatment (physical therapy adherence, bracing, participating in different sports that might advance Cobb angle progression, etc.). When analyzing the trendline in the control group, the trendline did have a negative slope of −0.7294 and an R-squared value at 0.112. This indicates that there is the greatest relationship with age and change in Cobb angle progression, but even then the relationship is weak if it is there at all. Due to the small sizes of the sampling groups, the internal and external validity of the results and of the graphs from the small R squared values indicate an extremely minimal relationship, if any, with age to the Cobb angle progression.

**Figure 11:**
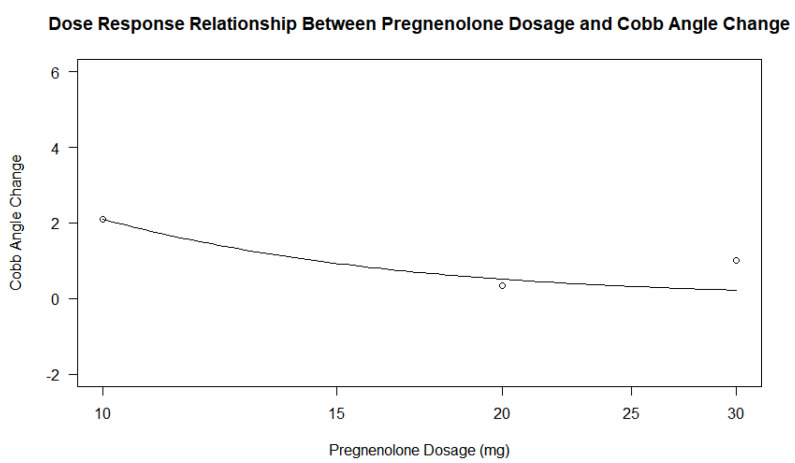
Dose–response relationship between pregnenolone dosage and cobb angle change treatment group only.

**Figure 12:**
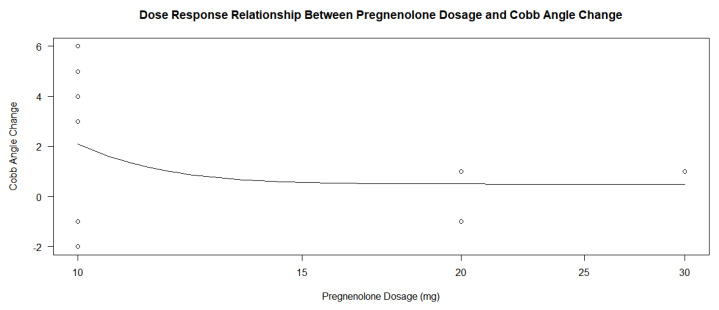
Dose–response relationship between pregnenolone dosage and cobb angle change treatment group only with all data points included.

The pregnenolone dose among the patients in the treatment group also varied slightly, ranging from 10 mg daily to 30 mg daily. A total of nine patients were prescribed 10 mg, three were prescribed 20 mg, and one was prescribed 30 mg. A dose–response analysis of the treatment group’s dosages found statistically insignificant *p*-values ranging between 0.7349, 0.9743, and 0.9555 when compared to differences in the Cobb angle measurements under three parameters. The dosage of the pregnenolone appeared to generally decrease the Cobb angle change, but the data points related to the 20 and 30 mg dosages did not show a significant difference. The small number of data points related to the higher dosages may contribute to the log-logistic analyses of the treatment groups under the three parameters giving high *p*-values. Figure 11 and Figure 12 show the scatterplots of the treatment group’s different dosages and their changes in Cobb angle using the averages of each dosage and each participant’s data, respectively. While there was a decrease in the overall response from the pregnenolone dosage, there were still some in the 10 mg group that showed greater improvement than the one taking the 30 mg dose. Another analysis was performed with all of the participants under the four parameters. The *p*-value was 5.039 × 10^−6^, with three of the parameters not giving information about the *p*-values. The statistically significant *p*-value gives extremely strong evidence that any form of pregnenolone treatment is beneficial to reducing or stabilizing Cobb angle change. However, as the dose in the treatment group alone did not produce significant *p*-values, this dose–response relationship is only upheld when the therapy is either not given at all or being given. In other words, there is extremely strong evidence for a statistically significant dose–response relationship when going from no pregnenolone supplementation compared to being treated with pregnenolone; however, this dose–response relationship is not seen between the different treatment groups. A difference was not found in Cobb angle progression between the 10 mg, 20 mg, and 30 mg treatment groups. As stated before, the small size of the treatment groups limits the internal comparison between the groups for a better understanding of dose–response relationship between groups taking the pregnenolone treatment. Further research is needed to uncover this relationship in greater detail. When taking the average change in Cobb angle in the different pregnenolone treatment dosages, the following values are obtained: 5.769 degrees for the 0 mg group, 2.100 degrees for the 10 mg group, 0.3333 degrees for the 20 mg group, and 1.000 degree for the 30 mg group. When looking superficially at the averages across the groups, it appears that any pregnenolone treatment does impact the curve progression in the participants.

From the ANCOVA tests run in R Studio to determine confounding variables, the presence of scoliosis at the start of the therapy was independent of the pregnenolone treatment, with a *p*-value of 0.414 [15]. Due to the high *p*-value, the impact that the initial curve had on the outcome was not related to the treatment of pregnenolone therapy. The ANCOVA type III test using the change in Cobb angle as the response variable, pregnenolone dosage as the treatment variable, and initial Cobb angle degree as the covariate yielded a *p*-value of 0.07074 for the pregnenolone dosage and 0.8560 for the initial Cobb angle. The initial Cobb angle is not statistically significant in this case while the dosage appears to have minimal effect on the change in Cobb angle with or without the initial Cobb angle degree. The initial Cobb angle’s large *p*-value suggests that it has a smaller and possibly no effect on the change in Cobb angle compared to the pregnenolone dosage. Initial Cobb angle appeared to have no effect on the change in Cobb angle later with or without the treatment. The continuation of Cobb angle occurred most prominently due to the pregnenolone dosage in this case compared to the initial Cobb angle. However, this relationship has weak evidence due to the *p*-value of 0.07074 when compared to these standards. The progesterone dose has a weak effect on the change in Cobb angle when controlling for the variable of age at diagnosis.

In the ANCOVA tests for the possible confounding variable of initial progesterone level in saliva, the progesterone level at the start of the treatment was independent of the pregnenolone dosage, with a *p*-value of 0.656 from the ANOVA test between the variables. The subsequent ANCOVA type III test with the change in Cobb angle as the response variable, pregnenolone dosage as the treatment variable, and initial progesterone levels as the co-variate yielded good evidence from the statistically significant *p*-value for the pregnenolone dosage at 0.007193, while the initial progesterone dose had an insignificant *p*-value at 0.5662. The progesterone dose has a statistically significant effect on the change in Cobb angle when controlling for the variable of initial progesterone levels.

## 4. Discussion

Pregnenolone therapy and its response effect on progesterone levels between the experimental and the control groups shows promising effects on the ability to increase salivary progesterone levels regardless of dose. The *p*-value of 0.00004739 provides extremely strong evidence that there is a profound relationship between the pregnenolone therapy’s ability to lead to a change in progesterone levels. As the average change was 37.64 after one year of treatment in the experimental group and 4.85 in the control group, the pregnenolone therapy led to a significant increase in salivary hormone levels in the patients. The 95% confidence interval in the treatment group’s analysis based on the null hypothesis that there would be no change in progesterone level was 25.73 and 49.55 with a *p*-value of 0.0000121. This large increase in the interval from 0 with a small corresponding *p*-value indicate that the treatment group showed extremely strong evidence that the pregnenolone therapy lead to an increase in salivary progesterone. The averages for the experimental groups across all dosages were much larger than that of the control group’s (34.80, 43.33, and 49, respectively, for the 10 mg, 20 mg, and 30 mg dosages). As mentioned previously, the highest change in salivary progesterone in the control group was 28, which was only larger than 21% of the experimental group participants’ individual numbers. While the *p*-values for the dose–response relationship for the pregnenolone therapy and the change in salivary progesterone were insignificant at 0.8300, 0.3259, 0.7877, and 0.9297, this was influenced by the fact that the dosage in the treatment group had no relationship. Through analysis of the two dose–response relationship graphs in Figure 5 and Figure 6 along with the average changes for each dose, there does appear to be a dose–response relationship. However, it is hard to obtain statistically significant *p*-values when any dosage in pregnenolone appears to increase the salivary progesterone near the same amounts. Since the treatment group accounts for a little over half of the doses, the drm() function in R has difficulty detecting this relationship that seems to be all or nothing with the pregnenolone dosage. Obtaining large *p*-values when comparing all the participants does yield *p*-values that were smaller in some cases (0.8300, 0.3259, 0.7877, and 0.9297) than the treatment groups’ *p*-values only (0.8738, 0.9812, and 0.8832). From the interpretation notwithstanding the *p*-values of the entire participant group, the conclusion that there is a relationship between pregnenolone therapy and its corresponding increase in salivary progesterone levels is obtained when analyzing the dose–response graphs of the entire group in Figure 5. However, the quantity of the dose has less overall impact on the change in progesterone levels compared to the relationship between not taking pregnenolone at all. From the results, a dosage of 10 mg appears to be almost as effective compared to the 20 mg and 30 mg dosages based on the large *p*-values alone. If seeking to increase salivary progesterone levels, a therapy of pregnenolone administered between 10 and 30 mg will yield increases in progesterone from the results above.

**Figure 13:**
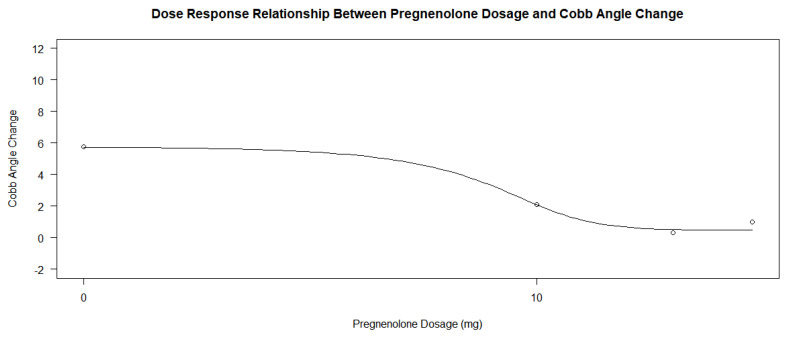
Dose–response relationship between pregnenolone dosage and cobb angle change across all participants.

**Figure 14:**
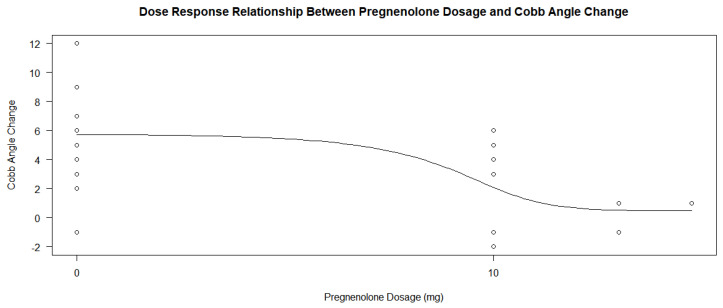
Dose–response relationship between pregnenolone dosage and cobb angle change across all participants with individual points.

**Figure 15:**
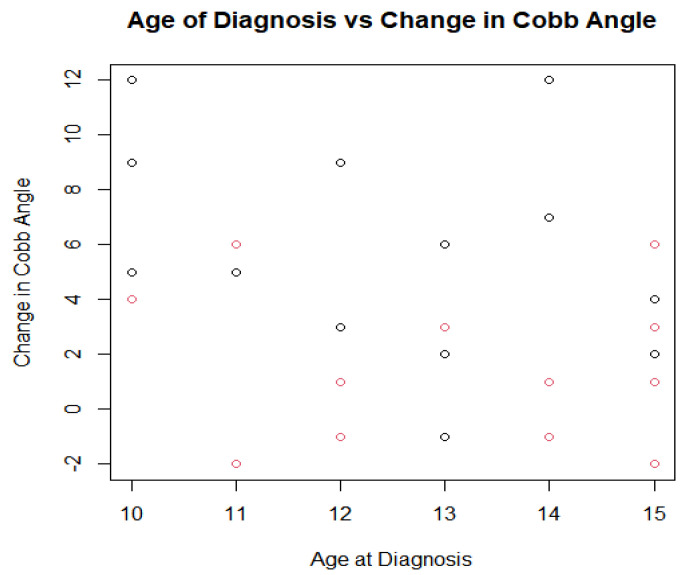
Age vs. change in cobb angle scatterplot (red dots indicate treatment group, black dots denote control group).

**Figure 16:**
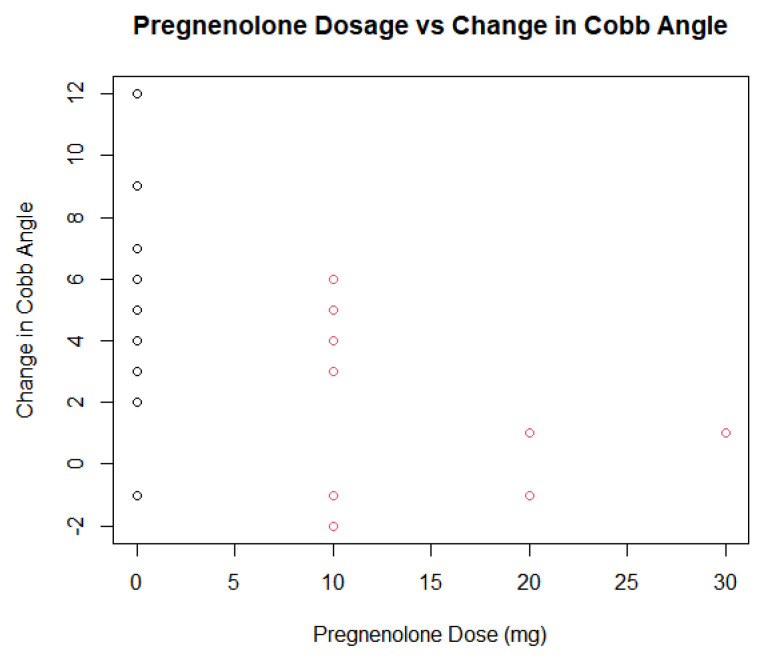
Pregnenolone dosage vs. change in cobb angle across all participants (red dots indicate treatment group and black dots indicate control group).

The analysis of two groups of consecutively selected scoliosis patient records showed that patients taking pregnenolone supplementation saw significantly less curve progression over 12 months when compared to scoliosis patients who did not take pregnenolone supplements as shown by Figure 13 and Figure 14 below. The small *p*-value of 0.005378 provides statistically significant evidence that the treatment group receiving the pregnenolone therapy experienced a smaller progression in their Cobb angles. The 95% confidence interval did not include zero, strengthening the argument that the treatment experienced a decreased curve progression compared to the control group. The two groups also showed significantly different salivary progesterone levels after 12 months. The age of onset was not strongly correlated to decreased curve progression. The minimal correlation coefficients between the patients’ Cobb angle change in relation to their age along with the large *p*-value of 0.4496 provides no evidence to support that the age of onset has an impact on the effectiveness of mitigating the change in Cobb angle. This holds true for both groups when analyzed separately as is shown in Figure 8, Figure 9, Figure 10 and Figure 15. Figure 16 specifically highlights both groups on the same plot separately, with the red dots indicating the treatment group and the black dots denoting the control group. The spread of the data indicated little correlation to age and change in Cobb angle, as some young and old participants showed similar changes in Cobb angle even when the groups were analyzed separately. Each of the trendlines had R squared values close to zero, with the largest being 0.112 for the control group. For the combined groups of all 27 participants, the curve progression (change in Cobb angle) tended to decrease as age increased; however, this was not the case for all of the points as shown in Figure 10 and Figure 15. There were some outliers for as high of an increase in Cobb angle in the 15 year old group, but the trend tended to show no decreases in Cobb angle depending on younger participant ages upon diagnosis. Compliance with other aspects of treatment (compliance with physical therapy routine, participating in different sports that might advance Cobb angle progression, etc.) may have also impacted the change in Cobb angle, which was not investigated in this study. When analyzing the trendline for the control group, the trendline did have a negative slope at −0.7294 with an R squared value of 0.112. This indicates that there is little relationship between age and curve progression, which is similar to the treatment and control groups that had R squared values of 0.0211 and 0.0727 respectively. Due to the low number of participants in the treatment group, it is difficult to generalize the results and compile a strong relationship between the age and Cobb angle change between the groups.

In reviewing the patient data, there were three instances of reported side effects, one for a patient who started on 30 mg of pregnenolone, and two who started on 20 mg of pregnenolone. Specifically, the side effects reported were mild breast tenderness, insomnia, and irritability. All of these side effects had resolved by a maximum of 8 weeks after initiating the pregnenolone. There were no other reported side effects.

Although the relationship between progesterone and scoliosis is unclear, this study is consistent with previous findings that female patients with scoliosis show significantly different levels of salivary progesterone compared to non-scoliotic control patients [16]. In adolescence, the chief roles of progesterone are to influence the development of long-term memory, including central pattern generators within the central nervous system [17], as well as mediate the signaling of peak bone mass development as a child nears skeletal maturity [18]. Since disturbances in both functions are more common in idiopathic scoliosis [19,20,21], continued investigations into more detailed associations between progesterone and idiopathic scoliosis are reasonable and warranted. The dosage did appear to minimally influence the decrease in curve progression within the treatment group, as seen in Figure 11 and Figure 12, but the small number of participants makes it difficult to extrapolate this trend to a larger population. The large *p*-values of 0.7349, 0.9743, and 0.9555 also suggest that higher dosages did have a strong effect on the change in Cobb angle.

### Limitations

Limitations inherent in the study design deserve consideration. While retrospective designs introduce the possibility of selection bias, this study aimed to mitigate this by inclusively selecting all patient records within a specific historical timeframe that met the inclusion criteria. However, it is crucial to note that male patients were excluded, thereby limiting the generalizability of the findings to females with a history of idiopathic scoliosis only.

The study involves a relatively small sample size, with only twenty-seven participants in total. If more data points or a stronger relationship could have been found, a more confident R-squared value could have potentially been calculated. From the results of this study, there is little evidence to support that there truly is a relationship between the age of onset and the curve progression. In Figure 8 of the treatment group’s graph, there are six points above the trendline, seven points below the trendline (two are at age 12 at −1), and one point on the trendline. In Figure 9 of the control group’s graph, there are six points below the trendline, one point on the trendline, and six points above the trendline with a high standard deviation. Similar results are present in the combined group’s trendline in Figure 10. There is a high standard error from the trendline, and a small R-squared equal to 0.0727. It is worth noting that the overall combined group experienced a negative slope at −0.6016. The graphs do not provide a strong relationship between the age of onset and the change in Cobb angle.

**Figure 17:**
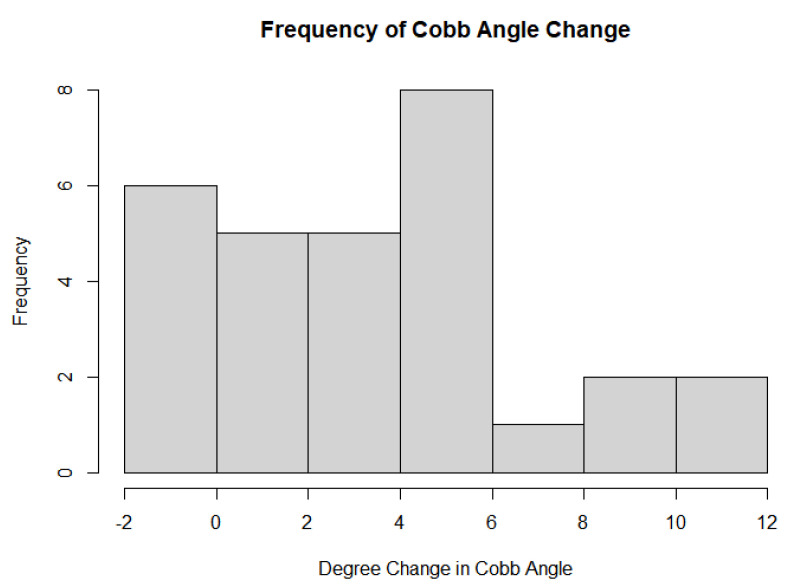
Histogram of change in cobb angle.

A further limitation to the study is the results from the normal distribution graph made in R-Studio. A histogram was used to plot the change in Cobb angle results as shown in Figure 17. The frequency was not normally distributed. Additionally, an asymptotic one-sample Kolmogorov–Smirnov test was used to analyze whether the change in Cobb angle was normally distributed. The *p*-value corresponding to the test was 0.00000000001253, indicating that the data are not normally distributed for the change in Cobb angle across all participants. From the linear analysis on the data, the following statistics are highlighted as follows. The formula for the linear regression (that corresponds with Excel’s values) is y = −0.6016x +11.25. The residual standard error is 3.891 on 21 degrees of freedom. The R-squared statistic is 0.0727 (which matches with the value calculated in Excel). The F-statistic is 1.003 on 6 and 21 degrees of freedom. Most notably, the *p*-value is 0.4496. Due to the extremely large *p*-value, there is very little evidence to support that the age of onset/diagnosis of scoliosis has an impact on the change in Cobb angle.

Concerning the observed Cobb angle differences, it is important to note that while the changes in Cobb angle were statistically significant, it is unknown based on the sample size and relatively small window of time as to whether these changes are clinically significant. Although the change may be within the margin of error for computerized Cobb angle measurements, it is possible that the magnitude of the Cobb angle change over a longer time period may show a larger Cobb angle change. This needs to be confirmed in future study.

Additionally, compliance with other scoliosis treatments like physical therapy exercises as well as how genetics influence the magnitude of curve development in each patient may have resulted in the outliers and weak correlation between age and Cobb angle development. Several patients in both groups were concurrently participating in some type of physical treatment for their curves. Some of the patients were prescribed bracing, some were performing a type of scoliosis-specific physiotherapy treatment like ScoliSMART, Schroth, or SEAS exercises, while others were on an observation plan. It is unknown how the impacts of these physical treatments impacted the current results. However, patients in both groups were participating in physical treatment, not just one group or the other.

Although the different doses of pregnenolone did not appear to change the Cobb angle values in the treatment group only, this may have been due to the low sample size of patients taking 20 or 30 mg of pregnenolone compared to the number of patients taking 10 mg. Figure 11 and Figure 12 show that there is a general decrease from the 10 mg to the 20 and 30 mg pregnenolone treatments, but the trend appears to plateau for the 20 and 30 mg pregnenolone therapy patients. When comparing the treatment group to the control group, they experienced overall a decrease in their Cobb angle change as shown in Figure 13, Figure 14 and Figure 16, with statistically significant *p*-values providing extremely strong evidence (0.000005039).

The insignificant *p*-values when comparing the treatment group to the dosage only do not show a relationship between pregnenolone dosage and Cobb angle change outcome, with *p*-values of 0.7349, 0.9743, and 0.9555 on log-logistic function with three parameters.

From the relationships analysis, clinical recommendations that can be extrapolated to female adolescent patients with low progesterone include to begin therapeutic treatment with pregnenolone. Recommendations for future studies include analysis of larger groups of patients within the pregnenolone treatment groups to provide a greater understanding of the dose–response relationship when therapy is administered.

It is also important to discuss the differences observed between Cobb angle progression among both groups. The treatment group progressed 1.64 degrees in one year, while the control group progressed 5.77 degrees. It is known that the risk of curve progression occurs chiefly between Risser stages 0–2, where bracing is typically recommended [1]. Morningstar also reported that scoliosis patients show lower salivary progesterone levels compared to non-scoliotic patients [16]. Progesterone serves to help regulate menstrual cycles, and perpetually lower progesterone can impact or delay menarche [22], which is a known risk factor for curve progression over a longer period of time [23]. From the results of this study, the administration of pregnenolone to patients resulted in increased salivary progesterone levels. Future research is needed to determine how the current results may be amplified if continued progression occurs over a longer time frame due to delayed skeletal maturation.

## 5. Conclusions

This study’s results suggest that female patients with a history of adolescent idiopathic scoliosis may benefit from pregnenolone supplementation. Patients taking pregnenolone were shown to experience higher salivary progesterone levels and smaller scoliosis curve progression after 12 months. Both of these results were significantly different when compared to a scoliosis patient control group that did not participate in the pregnenolone therapy. It is unclear whether the progesterone increase was related to the rate of curve progression; nevertheless, these results warrant further prospective investigation into this topic.

## Data Availability

The data presented in this study are available on request from the corresponding author. The data are not publicly available due to privacy restrictions.
